# Analysis of TLR7, SOCS1 and ISG15 immune genes expression in the peripheral blood of responder and non-responder patients with chronic Hepatitis C 

**Published:** 2017

**Authors:** Razieh Dowran, Jamal Sarvari, Afagh Moattari, Mohammad-Reza Fattahi, Amin Ramezani, Seyed Younes Hosseini

**Affiliations:** 1 *Department of Bacteriology & Virology, School of Medicine, Shiraz University of Medical Sciences, Shiraz, Iran*; 2 *Gastroenterohepatology Research center, Shiraz University of Medical Sciences, Shiraz, Iran*; 3 *Department of Medical Biotechnology, School of Advanced Medical Sciences and Technologies, Shiraz University of Medical Sciences, Shiraz, Iran*; 4 *Shiraz Institute for Cancer Research, School of Medicine, Shiraz University of Medical science, Shiraz, Iran *

**Keywords:** Hepatitis C virus, ISG15, SOCS1, TLR7, Responder

## Abstract

**Aim:**

To evaluate the baseline expression of the immune genes in PBMCs of responder and non-responder patients with chronic Hepatitis C.

**Background:**

Although the contribution of peripheral blood mononuclear cell (PBMC) gene expression in treatment outcome of hepatitis C virus (HCV) infection is supposed, it has remained to be distinctly delineated. The baseline expression of the immune genes inside PBMCs may reflect the responsiveness status following IFN treatment.

**Methods:**

Totally, 22 chronic HCV encompasses 10 responders and 12 non-responsive cases enrolled randomly regarding medical records. The PBMCs from the peripheral blood samples were isolated and then incubated for 6 hours in the culture media. The baseline expression of TLR7, SOCS1 and ISG15 was measured by Real time PCR.

**Results:**

The gene expression pattern in PBMCs of both groups showed a similar trend. The expression of SOCS1 and TLR7 genes showed higher levels in non-responder group (P>0.05). The result of ISG15 showed a higher but non-significant expression in the responder group (P>0.05).

**Conclusion:**

The similar pattern of TLR7, SOCS1 and ISG15 expression in the responder and non-responder patients indicated their poor discriminating and predictive value in PBMCs sample.

## Introduction

 As a growing health concern, hepatitis C virus (HCV) treatment is yet to be resolved in the medical settings. The HCV standard therapy encompasses pegylated interferon (peg-IFN) and ribavirin (RBV) associated with some shortcoming even after the adventure of new protease/polymerase inhibitors (1-3). As the main marker, the presence of virus genome 6 months after the end of treatment determines the therapy outcome and subdivides the patients into non-responder (NR) and responder with sustained virologic response (SVR). The variation in the genome of the virus in NR and SVR is one of the most important factors that influence the treatment outcome (4).

 The expression profile of some immune genes in the liver and peripheral blood mononuclear cells (PBMC) has shown differences in the setting of HCV chronic infection (2, 5). As an inquiry, whether deregulated baseline expression of innate immune genes is attributed to therapy responsiveness or clinical outcomes remains to be responded clearly (6). A couple of studies revealed significant differences in gene expression between NR and SVR groups in such a way that some are considered as predictive factors for therapy. Whereas liver remained the solely fertile ground for amplification of HCV, extra hepatic sources like PBMC have shown to have a special impact on virus maintenance and possible pathogenesis. However, hepatocyte and PBMC exhibit a nearly different profile of immune gene expression during the chronic HCV infection, indicating their different roles in disease progression (7-9). The data indicated that increased baseline of ISGs in NR patients is more significant in liver tissue than PBMC (10). Besides, the baseline immune gene expression correctly reflects responsiveness to therapy (11). Even though reports indicated antithetical achievements from gene expression in the liver and PBMC samples, peripheral blood remains an alternative sample reflecting the pattern of responsiveness to therapy. 

Several studies aimed to correlate PBMC gene expression pattern with the state of responsiveness (2, 5). While some indicated the predictive value of the immune genes of PBMCs as a marker, others acclaimed a poor prognostic value of them (2, 5, 10, 11). On the basis of the second group findings, ISGs expression levels in PBMCs did not show a significant difference between NR and SVR patients. 

The expression pattern of IFN related genes such as SOCS1, TLR7 and ISG15 genes was widely investigated as predictive factors of response (10-12). SOCS1 is the inhibitor of the JAK-STAT pathway. As SOCS1 has been assigned as a suppressor of cytokine signaling gene, its elevation in NR patients seems completely related to immune subversion and unresponsiveness (13). TLR7 is a member of the toll-like receptor (TLR) family. It detects single stranded RNA like HCV genome. It’s a vital component of antiviral immunity. Another gene investigated in this study was ISG15. It was identified as an interferon stimulated gene (ISG). Its expression is induced as a response to type I interferons and has anti-viral activity (14-15). Therefore, as ISG15 and TLR7 were attributed to IFN stimulation and induction pathways especially in HCV infected patients, their magnitude of expression may also influence the outcome of treatment. Regarding the need for predicting response in HCV patients, discrimination of NR and SVR groups by gene expression analysis of PBMCs seems to be worthy. Whether the expression level of the aforesaid genes in PBMCs harbors discriminative potency between responders and non-responders is controversial (14-18). Further investigation is required to clarify if the expression pattern of these genes is clearly different in SVR and NR groups in PBMCs. 

Here, we aimed to determine if baseline expression of the aforementioned genes was different among SVR and NR patients. 

## Methods

**Patients and samples**

Overall, 22 chronic HCV patients, including 12 non-responders (mean age 41 ± 9.6 years, range from 34 to 61) and 10 responders (40 ± 12.3 years old, range from 19 to 52) were admitted to Liver Clinic of Gasteroenterohepatology research center (Shiraz, Iran) and enrolled randomly in the project. The informed consent was obtained while ethical guidelines from Shiraz University of Medical Sciences were fully considered. Out of 12 non-responders, 9 patients were male and the other 3 were female. To the responder group, 8 males and 2 females were admitted. The mean age of SVR and NR patients was 42.9 and 50, respectively.

All the patients have received the standard peg-IFN alpha (180 mcg once weekly, subcutaneously) plus ribavirin (patient weight < 75 kg = 1000 mg, 5 tablets (2 morning, 3 evening) and <75 kg = 1200 mg 6 tablets (3 morning, 3 evening) therapy for 48 weeks since all of them were infected with genotype 1 of HCV. None of them consumed a new generation of Direct Acting Antiviral drugs. They were subdivided into responder and non-responder groups based on clinical records, detection of HCV RNA 6 months after the end of the treatment. All samples were taken from the patients infected with genotype 1 to achieve more homogenous results. Also, they were confirmed as negative cases of HIV/HBV infection. 

Blood samples were taken in heparin tubes and fresh PBMCs were isolated from blood samples by Ficoll-Hypaque (Inno-train, Germany) method. Then, PBMC samples were cultured in complete RMPI medium (with 10% bovine serum) in 6-well plates (106 cells/well) and incubated in the CO2 incubator for 6 hours. The cultured cells were then harvested and introduced into RNA extraction.

**Real-time PCR and gene expression analysis**

Total RNA was extracted using TRAZOL (YTA, Iran). The quantity and quality of the obtained RNA were checked by measuring the ratio of optical density (OD) of 260/280 nm using NanoDropTM (ND 2000c, Thermo) spectrophotometer and gel electrophoresis. About 600 nanograms of the total RNA was reverse transcribed using a RevertAid H-Minus First Strand cDNA Synthesis Kit (Fermentas, Lithuania). The expression pattern of TLR7, SOCS1 and ISG15 were measured using Real-time PCR assay. The expression level of each target gene was determined in duplicate and normalized to the expression level of human ACTB (actin, beta) reference gene. AlleleID software version 7 (Premier Biosoft, USA) was employed to design specific primers (Table 1). Real-time PCR was performed using an ABI Step Oneplus Sequence Detection System 2.1 (Applied Biosystem, USA). Finally, regarding the similar efficiency of different qPCR assays, 2-∆Ct formula was applied to determine the relative expression levels for genes (19). 

**Statistical Analysis**

 Data were expressed as the mean ± SEM and analyzed using Graph Pad Prism version 5 (San Diego, California). A P-value of less than 0.05 was considered to represent a statistically significant difference between the group means. The significance of the difference in the means was determined by Mann–Whitney’s U-test or unpaired t-test. 

## Results

**Figure 1:**
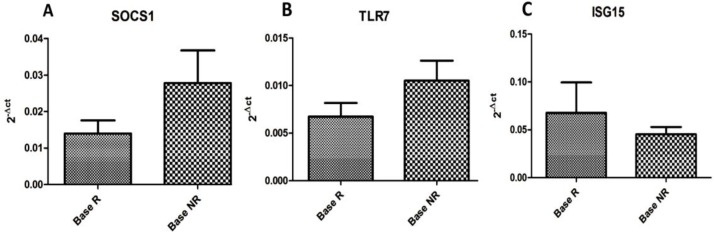
The expression level of SOCS1(A), TLR7 (B) and ISG15 (C) in PBMCs of the responder (Base R) and non-responder (Base NR) patient groups. Each bar is representative of the mean for each group sample ± SEM

The qPCR result of the expression pattern for 3 evaluated genes is shown in Figure 1. These results indicated that no significant difference was detected among the two groups. The expression level of SOCS1 in NR showed a higher level, but not in the significant level, as indicated in Figure 1-A (P>0.05). The SOCS1 gene correlates with an immune modulator role in IFN signaling and induction pathways. The expression pattern of TLR7 was similar to SOCS1 as revealed by higher expression in non-responders (P>0.05) compared to the responder group. It may indicate more inflammatory response among the non-responder group as this gene attributed to innate immunity and inflammation. In the case of ISG15, this pattern was completely subverted as the expression level was more in the responder patients. It was hypothesized that ISG15 reflects the state of the IFN signaling pathway and its integrity. Thus, the higher rate of ISG15 may indicate the better quality of IFN responsiveness paths in the responder patients as demonstrated in Figure 1C.

## Discussion

Due to the hardship of therapy for HCV, finding markers to discriminate between responder and non-responder cases before, during or after therapy is valuable. Although a bunch of findings is on the desk, attribution of the immune gene expression to treatment outcome is under investigation (7-11, 20). Studies demonstrating the expression difference between SVR and NR patients are frequent but drawing conclusive remarks, especially for PBMC samples, need further studies. However, these studies suggest that the basal gene expression and magnitude of its induction might predict responsiveness to the therapy (11).

Studies, which assessed gene expression in PBMCs of chronic hepatitis C patients are rare (20). In addition, while some indicated the predictive propensity of the immune genes of PBMCs, others acclaimed a poor predictive value for PBMCs as a reliable sample. Considering all the previous antithetical achievements, here we collected peripheral blood samples from NR and SVR patients and the baseline expressions of 3 different genes including SOCS1, TLR7 and ISG15 were compared (11, 18). 

**Table 1:** The list of primers employed in specific qPCRs

**Gene**	**Primers**
β-actin (ACTB)	Forward: 5′-GCCTTTGCCGATCCGC-3′ Reverse: 5′-GCCGTAGCCGTTGTCG-3′
TLR7	Forward: 5′-GAAAGTTGATGCTATTGGG-3′ Reverse: 5′-TTTGTCTCTTCAGTGTCC-3′
SOCS1	Forward: 5′- TTCGCCCTTAGCGTGAAGATGG-3′ Reverse: 5′- TAGTGCTCCAGCAGCTCGAAGA-3′
ISG15	Forward: 5′- TCATCTTTGCCAGTACAGGAGC-3′ Reverse: 5′- TTCTGGGTGATCTGCGCCTT-3′

In the case of SOCS1 expression, our data indicated an insignificantly higher level in the NR patients' group. The data may support the previous report regarding a similar pattern of expression in PBMCs among both SVR and NR groups (11). As SOCS1 has been assigned as a suppressor for IFN response pathway, its elevation seems to be conceptually related with unresponsiveness to IFN therapy, as described in non-responders (21). The SOCS1 gene correlates with IFN signaling pathway modulation (22, 23). The lower baseline expression of SOCS-1 was showed in the responder group while comparing the liver tissue. It was shown that SOCS-1 expression in PBMCs was predictive of treatment outcome in SVR (13). Greater expression of SOCS1 emphasizes its role as a suppressive agent, but it is useful just in liver tissues (21, 23-26). 

Baseline expression of TLR7 also is the same as that of SOCS-1. TLR7 expression analysis revealed that in the non-responder group, the amount of expression was higher (not significantly), compared to the responder group. The clue behind this result may arise due to inflammatory properties of this molecule (27-29). It may indicate more inflammatory response among the non-responder group, as this gene attributed in innate immunity and inflammation. The importance of TLR7 up-regulation in individuals who have progressed to the liver cirrhosis stage and unresponsiveness to IFN has been discussed before (30).

According to our result, TLR-7 expression may not be a reliable marker to discriminate between responsiveness and non-responsiveness. A report identified a reduced level of TLR7 in chronic HCV infected patients compared to healthy cases (31). Other results showed a significantly higher expression of TLR7 in the HCV infected samples when compared to uninfected controls (14). Boghdadi et al. reported that mRNA levels of TLR7 in monocyte cells was significantly higher in SVR compared to NR group; this supports its usefulness as a biomarker for IFN therapy (32). 

 ISG15 expression level has been suggested as a predictive factor of drug response when was measured in the liver (15, 16). Our results indicated that the baseline expression level of ISG15 in NR and SVR groups was nearly similar in PBMCs. The possible antiviral roles of ISG15 in the liver have been mentioned in the literature, but its exact role, especially in PBMC, has remained controversial and needs to be further investigated (15, 33). As it was hypothesized that ISG15 reflects the state of the IFN signaling pathway and its integrity, elicitation of higher ISG15 may indicate the better quality of IFN responsiveness (16, 33, 34). In the liver tissue, the baseline expression of ISG15 was determined to be signiﬁcantly lower in the responders, whereas in PBMCs, IFN related genes level was not signiﬁcantly different (7). In the other words, baseline expression patterns in PBMCs of the SVR patients resemble that of the NR group (11), a finding that was repeated in our experiment. 

Regardless of previous studies supporting our findings, the discrepancy between our findings and some of the others may be explained partly by differences in genetic background of the population, small sample size and even the genotype of the viruses. Hence, these results also indicated that measuring the baseline expression of ISGs in PBMCs may not be a suitable marker for response prediction and discriminating factor between SVR and NR patients, in contrast to those findings in the liver tissue (8).

While the presented study demonstrated an expression difference among the groups, it suffers from some limitations, including small sample size, few numbers of investigating genes and the absence of protein assessment. 

In conclusion, our data support the poor predictive and discriminating value of the baseline level of the aforesaid genes in the PBMCs samples for therapeutic approaches. Although the peripheral blood samples are the easiest one to be obtained, the measurement of baseline ISGs seems not to be a reliable approach used to predict the treatment outcome.
